# Global and Stage Specific Patterns of Krüppel-Associated-Box Zinc Finger Protein Gene Expression in Murine Early Embryonic Cells

**DOI:** 10.1371/journal.pone.0056721

**Published:** 2013-02-22

**Authors:** Andrea Corsinotti, Adamandia Kapopoulou, Carine Gubelmann, Michael Imbeault, Francesca R. Santoni de Sio, Helen M. Rowe, Yoann Mouscaz, Bart Deplancke, Didier Trono

**Affiliations:** 1 School of Life Sciences and Frontiers in Genetics Program, École Polytechnique Fédérale de Lausanne, Lausanne, Switzerland; 2 Swiss Institute of Bioinformatics, Lausanne, Switzerland; Colorado State University, United States of America

## Abstract

Highly coordinated transcription networks orchestrate the self-renewal of pluripotent stem cell and the earliest steps of mammalian development. KRAB-containing zinc finger proteins represent the largest group of transcription factors encoded by the genomes of higher vertebrates including mice and humans. Together with their putatively universal cofactor KAP1, they have been implicated in events as diverse as the silencing of endogenous retroelements, the maintenance of imprinting and the pluripotent self-renewal of embryonic stem cells, although the genomic targets and specific functions of individual members of this gene family remain largely undefined. Here, we first generated a list of Ensembl-annotated KRAB-containing genes encoding the mouse and human genomes. We then defined the transcription levels of these genes in murine early embryonic cells. We found that the majority of KRAB-ZFP genes are expressed in mouse pluripotent stem cells and other early progenitors. However, we also identified distinctively cell- or stage-specific patterns of expression, some of which are pluripotency-restricted. Finally, we determined that individual KRAB-ZFP genes exhibit highly distinctive modes of expression, even when grouped in genomic clusters, and that these cannot be correlated with the presence of prototypic repressive or activating chromatin marks. These results pave the way to delineating the role of specific KRAB-ZFPs in early embryogenesis.

## Introduction

About two thirds of the some 1500 transcription factors (TFs) encoded by mammalian genomes contain C2H2 zinc-fingers (ZF) allowing for sequence-specific binding to polynucleotidic sequences [1], [2]. Zinc-finger proteins (ZFPs) are found in yeasts and plants, but their diversity and complexity, notably reflected in the average length of their poly-ZF arrays, have steadily increased during evolution, suggesting that they were involved in speciation and the acquisition of higher functions [1]–[5]. More than half of human and mouse C2H2 ZFPs further harbor an N-terminal KRAB (Krüppel-associated box) domain constituted of 60 to 80 highly conserved residues conferring them with transcriptional repression potential. The KRAB domain is restricted to tetrapods, with the exception of one MEISETZ protein in sea urchin [1], [3], [5]–[8]. Some KRAB-containing proteins are devoid of ZFs, and are hence termed KRAB-O (KRAB-only), but still tend to be recruited to DNA through interactions with other TFs such as Sex Region Y (SRY) [9], [10].

KRAB-ZFP genes are often organized into clusters, with members sharing sequence similarity suggesting that they arose by endo-duplication from a common ancestor [5], [11]–[13]. Nevertheless, paralogous KRAB-ZFP genes also exhibit strong signs of positive selection, translating in the accumulation of non-synonymous mutations at positions encoding for the DNA-contacting residues of their ZFs, indicative of likely species-specific functions and engagement in genetic conflicts, as typically observed for genes encoding effectors of innate immunity [2], [5], [11]–[14].

Canonical KRAB-ZFPs and KRAB-O proteins likely share the ability to interact with the common cofactor KAP1 (KRAB-Associated Protein 1, also known as TRIM28 and TIF1β) [15]–[18]. KAP1 contains the canonical Ring, B-box and Coiled-Coil domains of RBCC proteins, in this case responsible for oligomerization and KRAB recognition [15]–[17], [19]–[23]. On the C-terminal side of the RBBC domain lies an effector region, involved in recruiting a set of heterochromatin-inducing factors such as HP1 (heterochromatin protein 1), the HDAC (histone deacetylase)-containing NuRD complex, and the histone methyl-transferase SETDB1 (also known as ESET), which mediates the tri-methylation of lysine 9 on histone 3 (H3K9me3). As a consequence, a commonly accepted model for KRAB/KAP1 action predicts that the sequence-specific docking of KRAB-ZFPs at given genomic loci can induce transcriptional repression, which can spread over several tens of kilobases, at least in somatic cells [20], [22]–[28].

The KRAB/KAP1 repression system plays essential functions during mouse development and in mouse embryonic stem cells (ESCs). KAP1 knockout embryos can progress through implantation but fail to gastrulate and undergo developmental arrest around day E5.5 [29]. *In vitro*, KAP1 depletion in ESCs by RNA-interference or Cre-mediated excision triggers a rapid loss of undifferentiated morphology, the down-regulation of pluripotency factors such as *Nanog* and *Oct4*, and the up-regulation of primitive streak markers and other lineage-specific genes, ultimately leading to cell cycle arrest and death [30]. An important indication that the KRAB/KAP1 system protects genome integrity during the early embryonic period was provided by the demonstration that KAP1 and the KRAB-ZFP ZFP809 are responsible for silencing murine leukemia virus and some other exogenous retroviruses in mouse ES and embryonic carcinoma (EC) cells [31]–[36]. KAP1 deletion was subsequently revealed to result in the transcriptional de-repression of a large set of endogenous retroelements, strongly suggesting that the control of these highly diverse and rapidly mutating genetic invaders may have been an important drive for the selection and evolution of KRAB-ZFP genes [31]–[36].

In spite of their numerical abundance and collective functional importance, it is remarkable that very few KRAB-ZFPs have so far been assigned specific functions. ZFP57 stands out, which was demonstrated to play an essential role in the maintenance of imprinting marks during early embryogenesis. *Zfp57*-knockout mice display broad alterations of genomic imprints, while mutations in human *Zfp57* correlate with transient neonatal diabetes mellitus, a disease associated with imprinting defects [37], [38]. Explaining these phenotypes, ZFP57 binds a methylated hexanucleotide present in all known imprinting control regions (ICRs), thereby recruiting KAP1, SETDB1 and DNA methyltransferases to these loci, which are then protected from the genome-wide wave of demethylation that takes place right after fertilization [39]. In addition, when KAP1 is depleted in murine maternal germ cells, the resulting heterozygous embryos display developmental defects probably in part due to altered maternal imprinting [40].

The present study examined the expression patterns of KRAB-ZFP-encoding genes during the early embryonic period. After establishing a census of genes encoding for KRAB-containing proteins (KRAB-ZFPs and KRAB-O) using the most recent releases of the Ensembl database, we measured their transcription in murine ESCs and other *in vitro* models of early developmental stages. This led to the identification of a subset of candidate genes, the expression of which correlates with pluripotency, matching chromatin-immunoprecipitation (ChIP) data with gene regulation mechanisms that might be involved in the control of KRAB-ZFP levels in embryonic stem cells.

## Results

### Updated Census of Murine and Human KRAB-containing Proteins

We first examined previous compilations of C2H2 ZFP genes, including KRAB-ZFP coding sequences [1], [5], [13], [41], [42]. Only two of these studies included murine genes [1] [41], whereas the rest focused exclusively on primates. However, we realized that both of these studies relied on genome releases dating back to 2009 at the latest, and we thus decided to generate an updated census of mouse and human KRAB-containing proteins basing on recent releases of the two genomes. Furthermore, we used Ensembl IDs for this purpose, first because in our experience it is less redundant than databases using RefSeq or gene name annotations, and second because we sought a system that could be used to cross data between databases and provide useful and unique information, such as chromosome position, gene sequence, details about protein-coding genes, etc.

**Figure 1:**
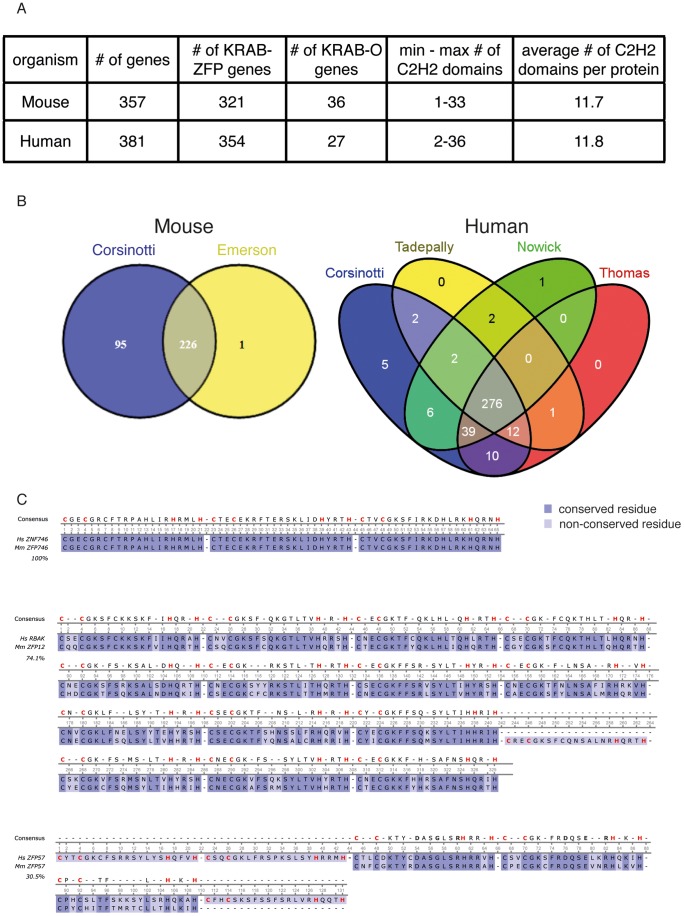
Mouse and human gene families encoding for KRAB-containing proteins and their conservation. A) Summary table of the mouse and human families. B) Venn diagram representing the comparison between mouse (left panel) and human (right panel) KRAB-ZFP gene lists generated in this work and in previously published studies [1], [5], [42], [48]. Numbers indicates the KRAB-ZFP genes identified in the different studies and the overlap among the different lists. C) Alignment of the C2H2 ZF domain amino acid sequences (excluding spacer sequences) of three mouse and human KRAB-ZFPs generated with Clustal Omega. Dark purple boxes indicate conserved residues. Light purple boxes indicate non-conserved residues. Dashes indicate gaps introduced by the alignment tool. Red residues highlighted in the consensus sequence point to the conserved cysteine and histidine residues of each C2H2 ZF domain. Human ZNF746 and mouse ZNF746 show 100% homology at the level of their C2H2 ZF domains. Human RBAK and mouse ZFP12 show 74.1% homology between their C2H2 ZF domains. Human ZFP57 and mouse ZFP57 show only 30.5% global homology between their C2H2 ZF domains, but the human third and fourth C2H2 ZF domains are homologous to the mouse first and second C2H2 ZF domains that are necessary and sufficient for the recognition of the conserved target sequence contained in ICRs [39].

We first updated the list of Ensembl mouse genes encoding for KRAB-ZFPs or KRAB-O proteins by interrogating four protein databases (http://pfam.sanger.ac.uk/, http://www.ebi.ac.uk/interpro/, http://smart.embl-heidelberg.de/ and http://prosite.expasy.org/scanprosite/) for accession IDs corresponding to the conserved structure of the KRAB domain (PF01352, IPR001909, SM00349 and PS50805, respectively), and using these as filters on the *Martview* tool of the *BioMart* project (http://www.biomart.org/biomart/martview/, dataset version GRCm37) to obtain a list of unique Ensembl Gene IDs. This led to the identification of 357 Ensembl-annotated KRAB-encoding genes in the mouse genome **(** **Figure 1a** **, Table S1)**. Based on the longest protein-coding transcript variants, 321 of them were predicted to encode for canonical KRAB-ZFPs, while the products of the remaining 36 did not contain any zinc finger and therefore corresponded to KRAB-O proteins **(** **Figure 1a** **, Table S1)**. The 321 KRAB-ZFPs harbored from 1 to 33 C2H2 zinc fingers, with an average of 11.7 such domains per protein **(** **Figure 1a** **, Table S1)**. Comparing our list with that previously obtained by Emerson and Thomas based on a 2009 genome annotation [1] revealed 95 previously not recorded protein-coding Ensembl KRAB-ZFP genes, while only one identified in this other study was missed by our approach **(** **Figure 1b** **, Table S2)**. Illustrating the utility of frequent updates in this area, ENSMUSG00000030424 (*Zfp939*) was not annotated as coding for a KRAB-containing protein when our list was generated (May 2012), but now it is. This comparison did not include KRAB-O encoding genes, as the previous study focused only on C2H2-ZFPs.

KRAB-ZFP genes are often found in clusters [1], [5], [12]–[14]. Using as definition a group of at least 2 genes within at most 200 kilobases from each other, we identified 50 KRAB-ZFP gene clusters in the mouse genome, some of which also comprised KRAB-O-coding genes. The largest cluster (number 3), located on chromosome 2, contained 41 genes. Cluster 49, on the X chromosome, contained only KRAB-O encoding genes, orthologous to the human Ssx group, associated with the t(X;18) chromosome translocation found in synovial sarcoma **(Table S1)** [43]–[46]. Of note, human *Ssx*-encoded KRAB-O proteins do not appear to interact with KAP1 [44]. We thus examined their mouse orthologs for the presence of aspartate and valine residues at positions 5 and 6 (D5, V6) of the KRAB domain **(Figure S1a)**, since this dipeptide motif was previously found to be necessary for KRAB-mediated repression and KAP1 recruitment, at least in the case of some KRAB-ZFPs [18], [47]. Based on alignments performed using the Clustal Omega online tool (http://www.ebi.ac.uk/Tools/services/web/toolform.ebi?tool=clustalo), we observed that, while D5 was present in all KRAB proteins, at least V6 was missing from all murine and human *Ssx* KRAB-O products, possibly explaining the lack of interaction between these SSX proteins and KAP1 **(Figure S1b and data not shown)**. However, the high degree of conservation of other residues in these proteins strongly suggests that Ssx-related KRAB domains must carry out KAP1-independent functions.

The functional conservation of TFs encoded by different genomes is often assessed by evaluating whether they recognize the same DNA sequences. In these terms, defining the functional conservation of KRAB-O genes is difficult due to their lack of DNA-binding domain and the high conservation of KRAB domains between species. However, the number and complexity of C2H2 domains represents a valuable tool for this kind of analysis in case of canonical KRAB-ZFP proteins. We therefore similarly generated a list of human KRAB-containing protein-encoding Ensembl genes. Out of 381 identified human genes, 27 encoded for KRAB-O proteins and the remaining 354 for canonical KRAB-ZFPs **(** **Figure 1a** **, Table S3)**. Based on the longest protein-coding transcript variants, human KRAB-ZFP proteins harbored between 2 and 36 C2H2 ZF domains, with an average of 11.8 **(** **Figure 1a** **, Table S3)**. When compared with lists of human KRAB-ZFP genes published in 2008 [5], and 2011 [42], [48], excluding KRAB-O coding genes, our census included an additional 62, 31 and 15 KRAB-ZFP genes respectively, of which 5 newly identified, and missed only 4 previously recorded members of this family **(** **Figure 1b** **, Table S4)**. Of note, the 4 genes missed by our approach were updated on the Ensembl database only after our analysis was completed (May 2012). While the discrepancy between the different censuses may partly stem from methodological differences, it stresses the importance of generating updated lists with each new genome release, in particular for large families of closely resembling genes.

We then defined mouse-human KRAB-ZFP putative orthologous pairs as displaying more than 70% amino-acid sequence homology in their C2H2 domains. This arbitrary threshold was defined with the aim of obtaining results that could be compared with previously published conservation analyses [1]. Only 117 such putative orthologous pairs could be identified, suggesting that a majority of KRAB-ZFPs accomplish species-specific functions **(Table S5)**. In some cases, such as for HsZNF354B, we observed matches with more than one putative mouse ortholog. This is due to the fact that different mouse proteins displayed >70% aminoacid conservation with the human protein at the level of their C2H2 domains. Even if it might be that only the most closely similar protein was the true ortholog, in these cases we included multiple matches in our analysis, per lack of functional data allowing for a more stringent selection. Among the most conserved KRAB-ZFP pairs, the C2H2 domains of HsZNF777 and MmZfp777 are 100% homologous, while for a markedly less conserved pair of putative orthogues, HsRBAK and MmZfp12, homology drops to 74%, albeit with 100% conservation at positions −1, +3 and +6 of the ZF’s alpha-helices, known to represent DNA contacting residues **(** **Figure 1c** **)** [1]. Noteworthy, our analysis did not identify human and murine ZFP57 as orthologous, despite formal evidence demonstrating that both regulate imprinting through recognition of the same DNA target [39]. Explaining this shortcoming of our approach, the two ZFs previously demonstrated as essential for recognition of the TGCC^m^GC methylated hexanucleotide present in ICRs are highly conserved between mouse and human ZFP57, yet these proteins harbor one and four additional non-homologous ZFs, respectively **(** **Figure 1c** **)**. Nevertheless, our data collectively support a model in which most KRAB-ZFPs are involved in species-specific functions, one of which, as previously demonstrated for KAP1 and ZFP809, is likely the control of host-restricted endogenous retroelements. Of note, sequentially diverse KRAB-ZFPs might be involved in a similar function, for instance if they control species-specific endogenous retroelements (EREs) located in the vicinity of a same gene. Indeed, the rewiring of functionally conserved core regulatory networks in ESCs could be correlated with TF binding sites located within species-specific transposable elements [49], and we found the KRAB-ZFP cofactor KAP1 to be essential for preserving the transcriptional dynamics of ESCs via the control of ERE-based enhancers [50].

### Expression of KRAB-ZFP Genes in Early Embryonic Cells

**Figure 2:**
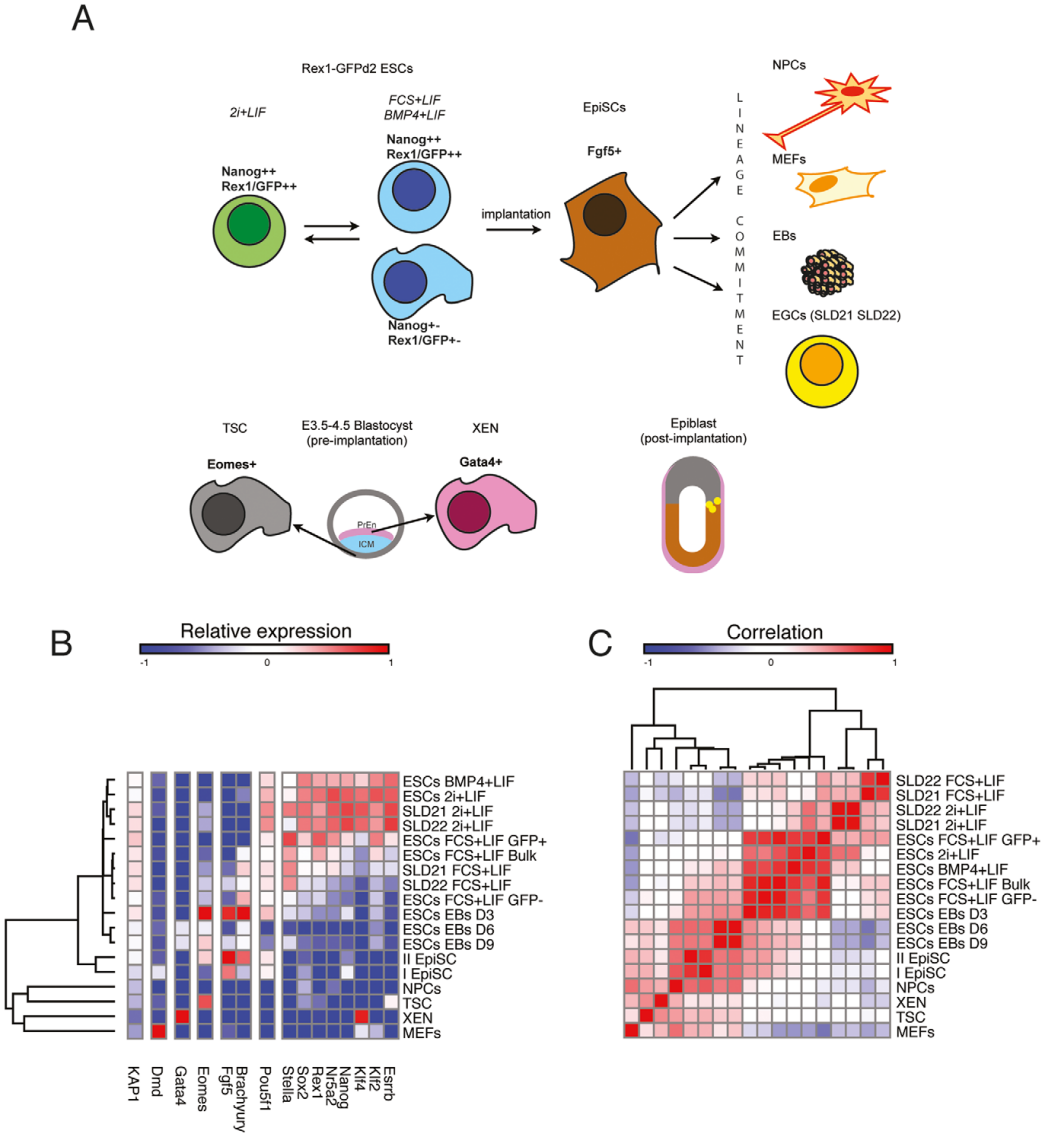
NanoString nCounter expression analysis in ESCs and other cell types. A) Schematic representation of the cell types corresponding to the different conditions used for the NanoString nCounter gene expression analysis, with corresponding developmental stages, culture conditions for ESCs, and expression of known marker genes highlighted. ESCs = Rex1-GFPd2 embryonic stem cells; EGCs SLD21 and SLD22 = two independently generated lines of embryonic germ cells; EpiSCs = epiblast stem cells; EBs = embryoid bodies collected at day 3, 6 and 9 of differentiation (D3, D6, D9); NPCs = neural progenitor cells obtained by direct differentiation of ESCs; MEFs = mouse embryonic fibroblasts; TSC = trophoblast stem cells; XEN = extra-embryonic endoderm stem cells. B) Heat map representing a clustering analysis of the different cell types based on relative NanoString counts (blue = lowly/not-expressed, red = expressed) of known marker genes for which a specific expression pattern between the different conditions is expected. C) Heat map representing a hierarchical clustering analysis and Pearson correlation (blue = low correlation, red = high correlation) of the different cell types basing on global expression values of KRAB-ZFP genes in the different conditions.

KAP1 is believed to act as co-repressor for a large number of KRAB-ZFPs and has been demonstrated through a number of independent observations to play essential roles in early embryogenesis [20], [29]–[37], [39], [40]. Yet whether and which KRAB-ZFPs partake in these processes remains largely unknown. As a first step towards addressing this issue, we assessed gene expression levels of Ensembl-annotated KRAB-ZFPs in a series of cells representative of the early embryonic period. For this, we designed a custom probe-set using the NanoString nCounter platform [51], [52]. Out of the 321 murine KRAB-ZFP Ensembl Gene IDs submitted to the manufacturer, 232 allowed the design of a specific nCounter probe covering all the transcript variants per gene, whereas the remainder exhibited too much sequence similarities for this task **(Table S6)**. As controls, probes to measure the expression of 21 housekeeping genes and 33 transcripts expected to yield specific expression patterns in the cell types under consideration were also included in the probe-set **(Table S6)**. We then selected a number of stem or differentiated cells commonly used as *in vitro* models for early mouse developmental stages **(** **Figure 2a** **)**, and subjected the corresponding RNAs to direct multiplexed expression analysis using the nCounter KRAB-ZFP probe-set **(Table S7)**.

To identify KRAB-ZFP genes, the expression of which is associated with pluripotent states, we grew mouse ESCs under several conditions (2i+LIF, BMP4+LIF and FCS+LIF). Furthermore, since in FCS+LIF, ESCs exhibit a significant degree of heterogeneity, with subpopulations expressing higher and lower levels of *Rex1* and other pluripotency markers such as *Nanog*, we used a transgenic line carrying a destabilized green fluorescent protein transgene under the control of the *Rex1* promoter (Rex1-GFPd2) to separate individual populations [53]–[55]. This allowed us to separately examine GFP^+^ and GFP^-^ cells in addition to the bulk population.

We then compared KRAB-ZFPs transcript levels in ESCs grown under these different conditions to those measured in: i) primary epiblast stem cells (EpiSCs) derived from the post-implantation epiblast and ESCs differentiated into EpiSCs and adapted to grow in the presence of recombinant FGF2 and Activin for 18 passages [56]; ii) ESCs differentiated into embryoid bodies (EBs) for 3, 6 and 9 days; iii) ESCs differentiated into neural progenitor cells (NPCs) [57]; iv) early-passage mouse embryonic fibroblasts (MEFs) [50]; v) trophoblast stem cells (TSCs); vi) extra embryonic endoderm stem cells (XEN) [58], [59]; and vii) two independently derived embryonic germ cell (EGC) lines (SLD21 and SLD22) cultured either in FCS+LIF or in 2i+LIF [60] **(** **Figure 2a** **)**.

To assess the quality of the nCounter probe set, we first performed a hierarchical clustering expression analysis of known control genes in the different settings **(** **Figure 2b** **)**. We could verify that expression of pluripotency markers (in particular, *Esrrb, Klf2, Klf4, Nanog, Nr5a2, Rex1, Sox2* and *Stella*) was associated with self-renewing ESCs/EGCs and was rapidly downregulated in differentiated cell types. In particular, higher expression of markers associated with ground-state pluripotency (*Nanog* and *Rex1*) was observed in ESCs/EGCs cultured in 2i+LIF compared with ESCs/EGCs grown in other conditions, and in the Rex1-GFP^+^ compared with Rex1-GFP^-^ ESCs. As expected, *Oct4* (*Pou5f1*) was expressed both in ESCs/EGCs and in EpiSCs, but not in the other cell types. Expression of the primitive streak markers *Brachyury* (*T*) and *Fgf5* was increased during early differentiation of ESCs into EBs and in EpiSCs. *Eomes*, a trophectoderm marker, was expressed in TSCs; similarly, *Gata4*, an endoderm marker, was expressed in XEN stem cells and in late EB differentiation (day 6 and 9). Expression of the Duchenne muscular dystrophy gene (*Dmd*) was detectable only in MEFs, which can be induced to differentiate into myotube-like cells under different conditions. Finally, KAP1 expression was sustained in all the different cell types, but was higher in undifferentiated cells compared with their differentiated counterparts **(** **Figure 2b** **)**.

Hierarchical clustering, using Pearson correlation as distance metric shows that global KRAB-ZFP gene expression level of EGCs and ESCs in self- renewing conditions clustered together **(** **Figure 2c** **)**. Early-differentiated EBs (day 3) also clustered with these two cell types, but this could be due to presence of incompletely differentiated cells in the early EB sample. Two other groups emerged from this type of analysis. The first encompassed EBs differentiated for 6 and 9 days and EpiSCs, and the second TSCs, XEN stem cells and ESCs differentiated into NPCs. Finally, global expression of KRAB-ZFPs in MEFs negatively correlated with all the other conditions.

**Figure 3:**
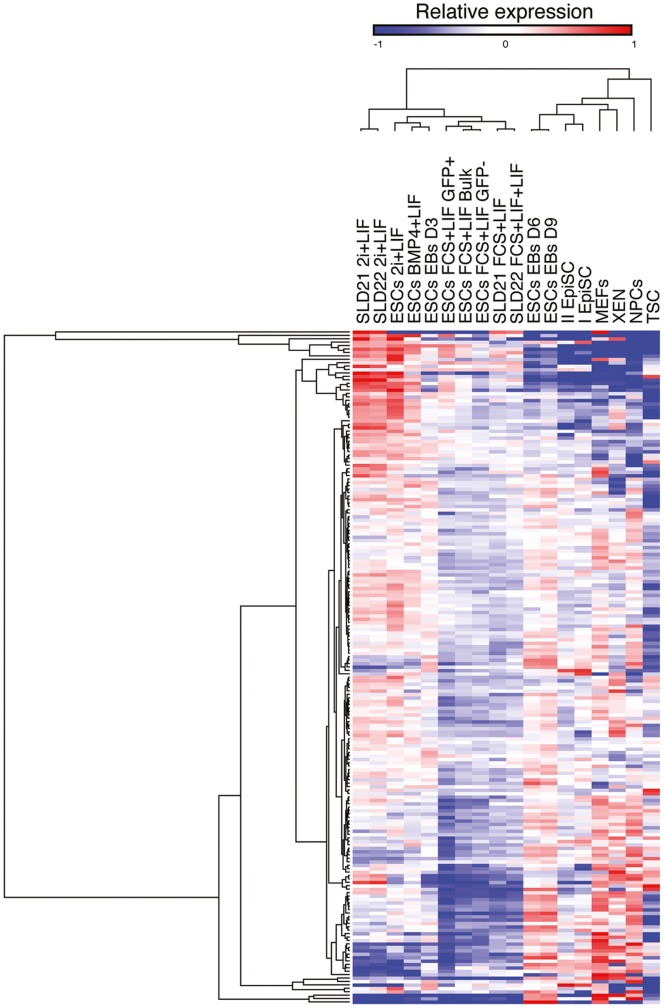
KRAB-ZFP gene expression analysis in ESCs and other cell types. Heat map representing a clustering analysis of the different cell types based on relative NanoString counts (blue = lowly/not-expressed, red = expressed) of KRAB-ZFP genes whose probes gave a signal above background level in at least one of the conditions. Each line corresponds to the signal derived from a single KRAB-ZFP probe.

Using as a reference the values obtained with control genes, the transcription of which or lack thereof had been extensively defined in previous analyses of the cells examined here, we set a threshold of 99 nCounter counts, above which we considered specific KRAB-ZFP genes as expressed. One hundred and sixty four probes gave a signal above this threshold in at least one of the cell types, but a significant fraction displayed cell-restricted expression patterns **(** **Figure 3** **and Table S7)**. In particular, some KRAB-ZFP genes gave a positive signal only in pluripotent cells grown under self-renewing conditions, and were down-regulated upon differentiation. Others presented an opposite pattern, with higher levels of expression in differentiated than in pluripotent cells, and some were unchanged between the various cell types. Finally, sixty-eight probes did not yield any signal in any of the cells analyzed here.

We then validated our expression data by RNA-seq. We compared the ratios between KRAB-ZFP gene expression signals in ESCs grown in FCS+LIF and MEFs in our dataset with the ratios obtained from previously generated RNA-seq data [31]–[36] in two independently generated ESC and MEF lines. In total, we were able to compare expression data for 181 KRAB-ZFP genes that were detectable in both the NanoString nCounter and the RNA-seq datasets **()**, for which we measured a positive correlation of 0.6431 with an R^2^ value of 0.5079 **(Figure S2)**. Furthermore, 36 of the 68 KRAB-ZFPs with NanoString scores below 99 in all tested conditions had RNA-seq counts in ESCs below 1, a value normally associated with genes silent in ESCs (data not shown). In sum, while the Nano-String approach seemed to be less sensitive for some lowly expressed KRAB-ZFPs, it also seemed reliable for our analysis.

### Identification of Stage-specific KRAB-ZFPs

**Figure 4:**
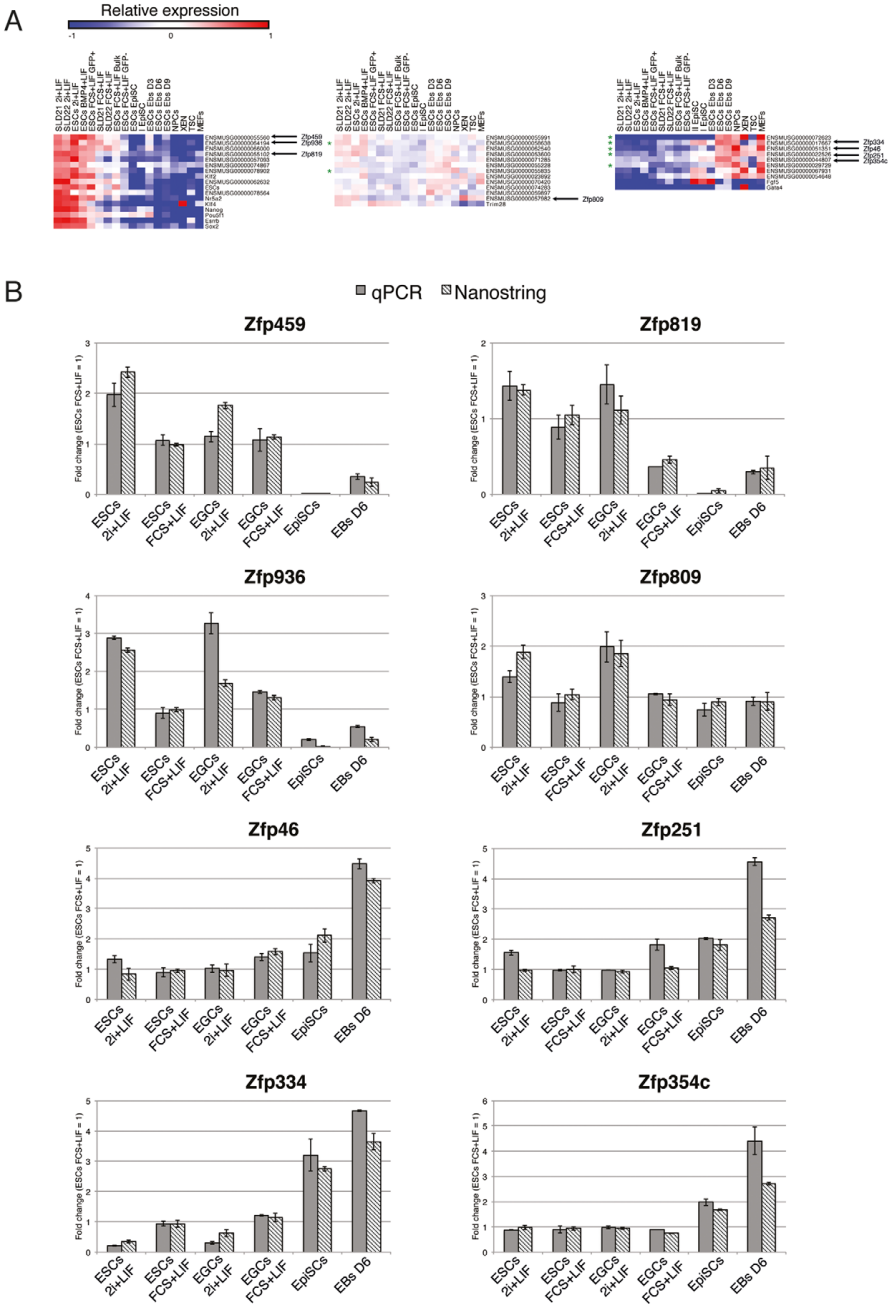
Identification of stage-specific KRAB-ZFP genes and validation by RT-qPCR. A) Heat maps representing relative NanoString counts (blue = lowly/not-expressed, red = expressed) of genes with expression significantly associated with undifferentiated pluripotent cells (up), with differentiated/adult cell types (bottom), or that are similarly expressed in all different cell types (housekeeping-like genes, center), based on a non-parametric t-test. KRAB-ZFP genes are denominated using Ensembl gene IDs. Control genes that belong to either one of the three categories are included using their gene symbols. Arrowheads indicate the Ensembl gene IDs of the subset of genes, the nCounter expression level of which was validated by RT-qPCR. Green stars indicate KRAB-ZFP genes that are conserved in human. B) NanoString nCounter vs RT-qPCR expression analysis of a subset of KRAB-ZFPs. Values indicate average fold change of the selected KRAB-ZFPs in pluripotent cells (ESCs 2i+LIF/FCS+LIF or EGCs 2i+LIF/FCS+LIF) and differentiated cells (EpiSCs and EBs D6), expressed in function of ESCs FCS+LIF (set as 1). Fold changes were calculated as the average of three independent experiments. Error bars represent standard deviation values (SD) over the three replicates.

We then divided the cells into 4 subgroups: ESCs and EGCs grown in 2i+LIF conditions; ESCs and EGCs grown in BMP4/FCS+LIF conditions; EpiSCs and EBs; XEN cells, TSCs, NPCs and MEFs. To identify genes with statistically significant expression levels specific for any of these groups, we performed a non-parametric analysis using the values obtained from each replicate of the cell types contained in the different groups. By setting the maximum adjusted p-value at 0.05 and by selecting genes with average expression levels that changed at least 2-fold between groups, we identified a subset of KRAB-ZFP genes, the expression of which was significantly associated either with undifferentiated pluripotent cells (grown in either 2i+LIF or in BMP4/FCS+LIF conditions) or with non-pluripotent cell, or was not significantly different between groups (housekeeping-like) **(** **Figure 4a** **)**. We identified 9 KRAB-ZFP genes associated with pluripotency, 8 significantly more expressed in non-pluripotent than in pluripotent cells, and 12 with a housekeeping-like mode of expression. Based on previously described criteria for the identification of mouse-human orthologs, none of the pluripotency-associated genes had a predicted human ortholog, compared with 5 out of 8 and 2 out of 12 for the second and third groups, respectively **(** **Figure 4a** **, green stars; Table S5)**. We then selected a subset of genes belonging to these three categories for real-time quantitative PCR (RT-qPCR) validation of the nCounter expression data **(** **Figure 4a** **, arrowheads)**. We measured the expression of these genes in ESCs/EGCs grown in 2i+LIF and FCS+LIF, in primary EpiSCs and in day 6 EBs. When normalized to ESCs grown in FCS+LIF, expression of these genes correlated between levels achieved using the nCounter platform and RT-qPCR **(** **Figure 4b** **)**. The results of the RT-qPCR confirmed that *Zfp459*, *Zfp819* and *Zfp936* were specifically expressed in self-renewing pluripotent cells, consistent with their rapid silencing upon differentiation **(Table S7)**. RNA levels of *Zfp809*, the KRAB-ZFP responsible for restricting MLV in murine ES and EC cells, did not significantly differ between the tested conditions, whereas *Zfp334, Zfp46, Zfp251* and *Zfp354c* were predominantly expressed in differentiated cells **(** **Figure 4b** **)**. Based on our definition of KRAB-ZFP gene clusters, *Zfp459, Zfp819* and *Zfp936* are located in KRAB-ZFP genomic clusters 40, 23 and 22 respectively; *Zfp809* in cluster 30; and, *Zfp334, Zfp251, Zfp354c* in clusters 2, 42 and 33, respectively, while *Zfp46* does not belong to any gene cluster **(Table S1)**.

### Regulation of KRAB-ZFPs in Mouse Embryonic Stem Cells

Mammalian transcription units grouped in genomic clusters, such as homeobox or olfactory receptor genes, are often regulated through interdependent mechanisms. While little is known about the transcriptional regulation of KRAB-ZFPs, it was previously reported that KAP1 binds the 3′ end of a subset of these genes in two somatic cell lines, suggesting some sort of auto-regulatory loop. Confirming previously reported data [13], we observed that the expression of specific KRAB-ZFP genes and changes thereof was independent of their chromosomal location, and that members of the same genomic cluster were independently regulated. Interestingly, we observed that KRAB-ZFP genes found in a same cluster could not only be differentially expressed within a given cell type, but also differentially regulated in a dynamic system, such as in differentiating ESCs (see for example Zfp459 and Zfp819 expression levels compared with those of the other genes grouped in the same clusters) **(Table S7)**. To expand our analysis, we explored a possible correlation between expression of KRAB-ZFP genes in mouse ESCs and histone marks within a region extending from 3.5 kb upstream to 500 bp downstream of their transcriptional start site (TSS), defined as gene promoter. Using available ChIP-seq data [39], [50], we focused on tri-methylated histone 3 lysine 4 (H3K4me3) and tri-methylated histone 3 lysine 27 (H3K27me3), which are typically associated with transcriptional activation and repression, respectively, and tri-methylated histone 3 lysine 9, a frequent signature of KAP1-mediated silencing, and matched these parameters with KAP1 ChIP-seq binding data. H3K4me3 was present at 189 KRAB-ZFP gene promoters, whereas only 25 bore the H3K27me3 mark, in 12 cases together with H3K4me3, a “bivalence” suggesting that they were poised for transcription **(Figure S3)**. However, no clear correlation could be established between the presence of any of these histone marks and the levels of expression of the corresponding KRAB-ZFP gene (not illustrated). Furthermore, some KRAB-ZFP genomic clusters, such as cluster 3, were globally depleted of both H3K4me3 and H3K27me3, whilst others, such as clusters 5 or 19, bore exclusively one of the two marks **(Figure S3)**. KAP1 was bound at a single KRAB-ZFP gene promoter, whereas 74 of them carried the H3K9me3 mark **(Figure S3)**.

**Figure 5:**
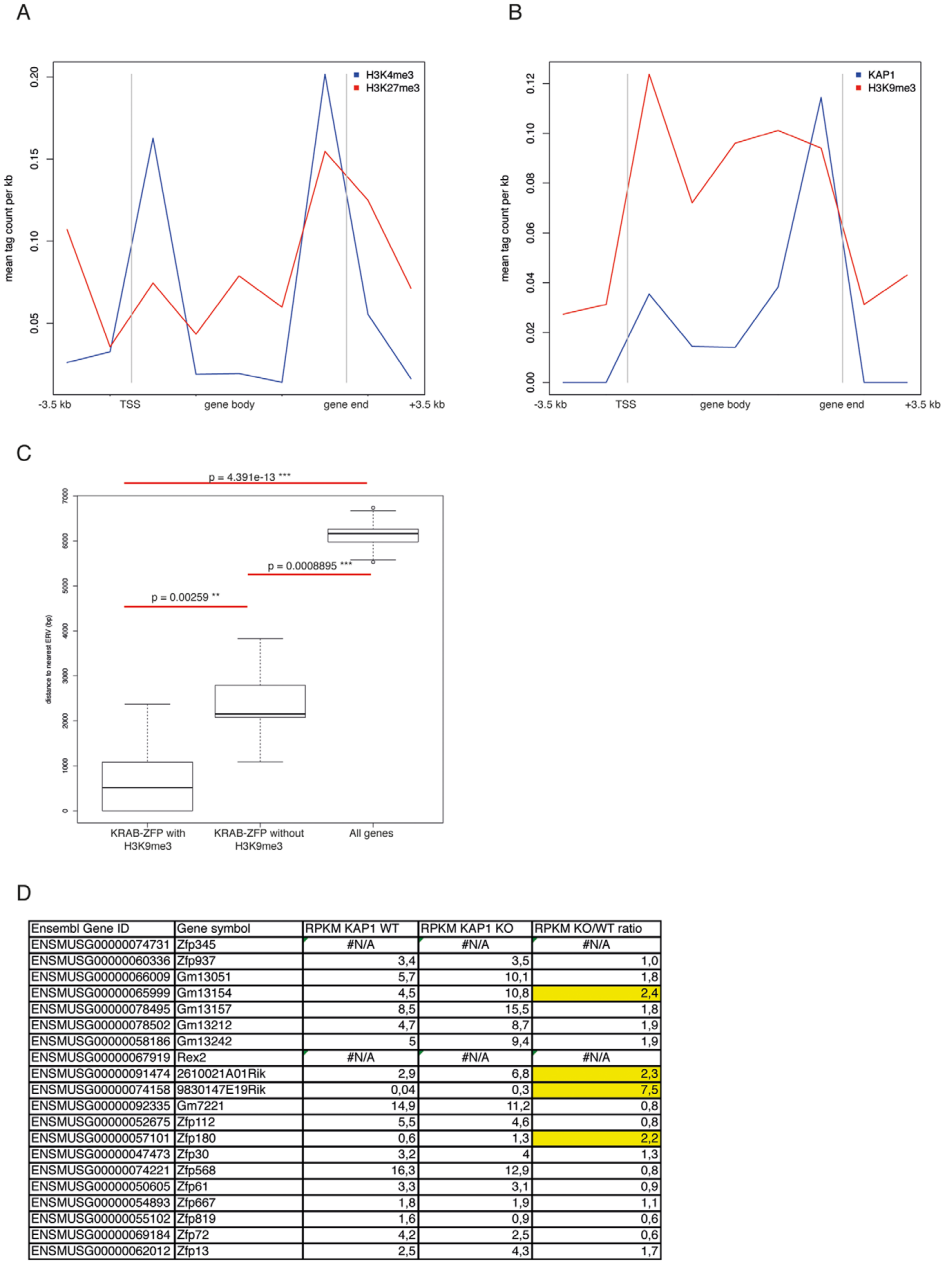
Distribution of histone-modifications and KAP1 binding over KRAB-ZFP gene bodies and surrounding genomic regions. The genomic regions contained between the TSS and gene end of mouse KRAB-ZFP genes, plus the regions including 3.5 kb upstream of the TSS and 3.5 kb downstream of the gene were equally divided in 8 “bins”. Identification of enriched sequences for H3K9me3, H3K27me3, H3K9me3 and KAP1 was performed using publicly available ChIP-seq datasets generated in mouse Rex1-GFPd2 ESCs (H3K4me3, H3K27me3, H3K9me3 [55]) and in KAP1 WT ESCs [39]. For details, see Methods section. A) Distribution of H3K4me3- (blue track) and H3K27me3- (red track) enriched regions over targeted KRAB-ZFP genes. B) Distribution of KAP1 (blue track) enriched regions over targeted KRAB-ZFP genes, highlighting the preferential enrichment for KAP1 downstream of the TSS with no spreading over the rest of the genes, as previously described [21], [61], while H3K9me3 (red track) spreads over the gene body up to downstream of the TSS. C) Distance between KRAB-ZFP genes enriched for H3K9me3 in ESCs and nearest ERVs compared with the distance of other KRAB-ZFP genes and other genes. P-values were calculated using a Wilcoxon test. D) Table summarizing RNA-seq RPKM counts in WT and *Kap1* KO ESCs and ratio KO/WT [31] for the 20 KRAB-ZFP genes with KAP1 enrichment in the regions contained between 3.5 kb upstream of the TSS and 3.5 kb downstream of the gene end. “N/A” indicates that no reads were detected for the corresponding gene. Highlighted in yellow are genes up-regulated more than 2 folds upon *Kap1* KO.

We then extended our analyses to the entire body of KRAB-ZFP genes, including 3.5 kb upstream of their TSS and 3.5 kb downstream of their gene end coordinates. H3K4me3 (207 genes) was generally enriched immediately downstream of the TSS and upstream of the gene 3′ end, but depleted in the central part of the transcribed region. While some H3K27me3 (44 genes) was found upstream of the TSS, this mark was surprisingly more frequently enriched towards the 3′ end **(** **Figure 5a** **)**. Only 20 KRAB-ZFP genes bore any KAP1; in these cases, it was most often bound towards their 3′end, as previously observed in somatic cells [61]. In contrast, H3K9me3 (181 genes, including 15 of the 20 KAP1 targets) was deposited all over the gene body, although its levels dropped markedly before reaching the TSS **(** **Figure 5b** **)**. Interestingly, the distance between 181 KRAB-ZFPs that bore H3K9me3 on their genomic regions and the nearest ERV sequence was significantly lower than for remaining KRAB-ZFP or other genes, suggesting some spreading of the repressive mark from ERVs into the body of these genes **(** **Figure 5c** **, Table S9)**.

To ask whether KAP1 regulates the transcription of KRAB-ZFP genes, we examined RNA-seq data generated in *Kap1* WT and *Kap1* KO ESCs [31]. Of the 20 KRAB-ZFPs enriched for KAP1, 18 displayed mRNA levels detectable in both control and *Kap1*-deleted cells. However, only 4 of them, *2610021A01Rik*, *9830147E19Rik*, *Gm13154* and *Zfp180*, were up-regulated more than 2-fold upon KAP1 removal, while the remaining 14 were either unaffected or down-regulated, including *Zfp72* (ENSMUSG00000069184), the only KRAB-ZFP bearing KAP1 at the promoter **(** **Figure 5d** **, Figure S3)**.

We then focused on the three pluripotency-associated KRAB-ZFPs, for which we had validated RT-qPCR expression data in different conditions **(** **Figure 4b** **)**. We observed that none of the examined histone marks distinguished them from other KRAB-ZFPs, including ones located in the same genomic clusters **(Figure S3)**. We then examined their promoters for the recruitment of TFs assigned to the core pluripotency network (C-MYC, E2F1, ESRRB, KLF4, NANOG, NMYC, OCT4, SMAD1, SOX2, STAT3, ZFX), as reported in previously published ChIP-seq data [62]. Interestingly, for *Zfp459* (bound by E2F1, ESRRB, KLF4, NANOG, SMAD1, SOX2, STAT3) **(Figure S4a)** and *Zfp819* (bound by E2F1, ESRRB, KLF4, NANOG, SOX2) **(Figure S4b)**, but not for *Zfp936* (not illustrated), we qualitatively observed an increased enrichment for core pluripotency network TF binding sites, unlike any other gene in the same genomic clusters.

## Discussion

KRAB-ZFPs represent the largest family of TFs encoded by tetrapods, yet only very few of them have been extensively characterized [1], [2], [5], [13]. Nevertheless, KRAB-ZFPs likely accomplish many of their functions through their cofactor KAP1, which has been demonstrated to play critical roles during early mouse development and in ESCs [8], [29]–[33], [35]–[40]. As a first step towards investigating the involvement of specific KRAB-ZFPs in these effects, we explored the expression and regulation of this gene family in a series of cells representative of mouse early developmental stages.

When this project was initiated, KRAB-ZFP genes annotation was incomplete, in particular for the mouse genome, partly because available lists relied on outdated genome releases. We thus proceeded to an updated census of murine and human KRAB-containing proteins, using Ensembl-annotated protein-coding sequences as a basis and the latest releases of either genome, which led to the identification of several tens of previously unrecognized KRAB-containing proteins. In the mouse, we identified 357 genes that could encode for these factors, 321 of which were predicted to be responsible for canonical KRAB-ZFPs and the remaining 36 for KRAB-O proteins. These KRAB-encoding genes are organized on chromosomes either as singletons or in 50 genomic clusters, defined as the occurrence of at least two members of the family within 200 kb. This observation is consistent with the proposal that C2H2 TF genes evolved by endo-duplication starting from a common ancestor, followed by functional divergence [1], [3]. Evolutionary studies suggest that the first KRAB domain linked to C2H2 zinc-fingers occurred in the *Meisetz* gene of sea urchin, but that the KRAB-containing gene family expanded only after the segregation of tetrapods from lower vertebrates [1], [6], [7]. The sporadic occurrence of KRAB-O encoding genes within clusters of canonical KRAB-ZFP genes **(Table S1)** suggests that these two classes of proteins co-evolved, and that the KRAB-O group might have arisen through the loss of the ZF-coding region of KRAB-ZFP genes. In some cases, KRAB-O proteins mediate locus-specific transcriptional repression through interaction with other TFs such as SRY [9], [10]. However, a whole cluster of KRAB-O proteins, encoded by the *Ssx* locus of both mice and humans, does not appear to interact with KAP1 [44]. Explaining this observation, we found that the KRAB domain of Ssx KRAB-O proteins is systematically mutated in one of two amino acids (D5, V6) that are normally conserved in KRAB-domains and that were previously demonstrated, at least in the case of some KRAB-ZFPs, as necessary for KRAB-mediated repression and KAP1 recruitment [18], [47]. The absence of V6 from Ssx proteins could be a potential explanation for their inability to interact with KAP1, which is consistent with the hypothesis that these proteins might have evolved a KAP1-independent function [46]. While this observation adds an extra layer of complexity to the study of KRAB-O-mediated functions, the high similarity of the amino acidic sequences of these proteins (not illustrated) further complicates their evolutionary analysis, so that we excluded them from our subsequent investigation.

In the human genome, we identified 381 genes encoding for KRAB-containing proteins, including 354 for canonical KRAB-ZFPs and the remaining 27 for KRAB-O proteins. One of the parameters that can be exploited to define the conservation of a TF between different species is the ability to bind the same DNA sequence. We therefore aligned the C2H2 ZF sequences of each mouse and human KRAB-ZFP and, by setting a threshold of 70% homology that allowed us to score most of the previously described ortholog pairs [1], we identified 117 putative orthologous pairs. This corresponds to less than one third of either family, consistent with the previously stressed evolutionary divergence of C2H2 ZFP genes [1], [3], [5]. This method also led to the identification of multiple putative ortholog pairs, with more than one candidate displaying more than 70% aminoacid identity within its C2H2 domains with a protein from the other species. In these cases, the lack of functional data precluded a more stringent designation of the “true” orthologs. Interestingly, while our approach easily detected orthologs proteins sharing high homology over their entire DNA-binding domains, it had limitations. It indeed failed to identify ZFP57 as one such protein, whereas we and others previously demonstrated that it is involved in the control of genomic imprinting in both species through the recognition of a same ICR-contained methylated hexanucleotide [37]–[39]. Explaining this shortcoming of our *in silico* approach, ICR recognition is mediated by two of the three ZFs present in mouse ZFP57, which although conserved in human ZFP57 represent only 2 out of the 6 ZFs present in this protein, so that aligning the DNA-binding domains of the two ZFP57 orthologs yields less than 70% homology. Whether other mouse-human orthologous pairs go undetected because of this phenomenon remains to be defined, together with the roles of ZFP57 zinc-fingers not involved in ICR motif recognition.

We then generated a cartography of KRAB-ZFP gene expression in pluripotent cells and other cell types commonly used as *in vitro* models of mouse early developmental stages. We selected a large variety of cells including pluripotent ESCs and EGCs grown in different self-renewing conditions and differentiated to other cell types, as well as other primary cells obtained from extra-embryonic tissues and from mouse embryos after implantation. Due to the complexity arising from the high degree of sequence similarity between members of the KRAB-ZFP gene family, we designed a customized NanoString nCounter probe set in order to obtain the proper sensitivity and specificity for direct multiplexed measurement of mouse KRAB-ZFP mRNA levels [51], [52], [63], [64]. While specific probes could be designed for only 232 KRAB-ZFP genes, owing to the high degree of sequence similarity between subsets of the remaining members of the family, our semi-quantitative analysis revealed that 164 of them were expressed above background levels in at least one of the cell types under study. A comparison between RNA-Seq and Nano-string data in ESCs and MEFs indicated that most KRAB-ZFPs scoring below threshold in this analysis were expressed either very lowly or not at all. We also observed a positive correlation between the two techniques, although with a relatively low correlation coefficient that could be explained at least in part by the distinct cell lines used to generate the datasets and by the different material analyzed (total RNA for NanoString and cDNA libraries for RNA-seq). An interesting degree of cell-specificity was noted in the levels of some KRAB-ZFP transcripts, so that patterns emerged that were proper to particular developmental stages, for instance pluripotent cells versus cells isolated after implantation or lineage commitment. After verifying the expression levels of a subset of candidate genes by RT-qPCR, we identified *Zfp459*, *Zfp819* and *Zfp936* as specifically expressed in pluripotent cells. Supporting a link between *Zfp459*, *Zfp819* and pluripotency, two recent reports demonstrated that expression of *Zfp459* is induced during the late stages of reprogramming MEFs into induced pluripotent stem cells (iPSCs) [65], and that the promoter of *Zfp819* is bound by OCT4, KLF4 and SOX2 between a pre-iPSC stage and fully reprogrammed iPSCs [66]. Interestingly, when we sought potential transcriptional regulators of these three pluripotency-associated KRAB-ZFP genes using publicly available ChIP-seq data, we noticed that the genomic regions encompassing *Zfp459* and *Zfp819* gene bodies and flanking sequences were enriched in binding sites for TFs belonging to the core pluripotency network, and that this phenomenon was specific for these two genes compared to other transcription units contained in the same genomic clusters.

In that view, it may seem surprising that none of the pluripotency-associated murine KRAB-ZFP genes was conserved in human, while several of their differentiated cell-specific or housekeeping-like counterparts had readily identifiable orthologs. However, one of the best established functions of the KRAB/KAP1 system is the silencing of endogenous retroelements during the early embryonic period [31]–[36]. As a large fraction of these genetic invaders, which contain *cis*-acting regulatory elements that can affect neighboring genes [49], are species-restricted, it is expected that their silencing in different species require distinct sets of sequence-specific repressors. Of note *Zfp809*, previously demonstrated as responsible for repressing MLV through recognition of the sequence encoding for its primer-binding site [31]–[36], did not exhibit a pluripotency-restricted expression pattern, consistent with the detection of its anti-retroviral activity in at least ES and EC cells. More generally, we hypothesize that the positive selection of KRAB-ZFP genes was in large part driven by waves of invasions by rapidly mutating retroelements, a phenomenon that is still active at least in a majority of species, thus explaining the emergence of a set of species-restricted TFs recognizing distinct sequences yet all aimed at the same goal, namely, preserving the transcription dynamics of early embryos through the silencing of retroelements.

We completed our study by examining mechanisms possibly responsible for controlling KRAB-ZFP gene expression. We first observed that expression of individual KRAB-ZFP genes usually did not correlate with that of their chromosomal environment, and that, within a same cluster, adjacent genes usually exhibited greatly different expression patterns, as previously demonstrated [13]. We also observed that this was true not only within the same cell type, but differential regulation of KRAB-ZFP genes also happened in the transition between different cell types, such as in the case of differentiating ESCs. Two typical examples of this behaviour were *Zfp459* and *Zfp819*, the pluripotency-restricted expression of which was unique amongst members of the genomic clusters in which they resided. We further observed that the promoter of most KRAB-ZFP genes was enriched in the activation histone mark H3K4me3 and depleted in its repressive counterpart H3K27me3, which was instead found towards the 3′ end of a subset of these genes, but that the presence of either of these chromatin modifications did not correlate with expression. Even if the promoters of the KRAB-ZFP genes found in cluster 5 and 19 were almost homogeneously loaded with H3K4me3 or H3K27me3, respectively, suggesting pan-cluster transcriptional regulation, expression levels of individual genes within these units did not fulfill this prediction. Interestingly, when we looked at the global deposition of these two histone-modifications over whole KRAB-ZFP gene bodies and the surrounding 3.5 kb, we observed that H3K27me3 was mainly enriched in the 3′ end region of the genes, pointing to a role distinct from promoter modulation; H3K4me3 was instead enriched both downstream of the TSS, suggesting a direct role in the modulation of promoter activity, but also at the 3′ end of the genes, similarly to H3K27me3, a phenomenon that had not been previously noted [67]. As well, even if H3K9me3 deposition was commonly found on KRAB-ZFP gene promoters, only one of them was enriched for KAP1. However, KAP1 was found in the body of 20 KRAB-ZFP genes, most often close to their 3′ end as previously described [61]. This supports the previous hypothesis that KAP1 does not always act as a transcriptional repressor, notably on KRAB-ZFP genes [68], [69]. In contrast, H3K9me3 seemed to spread from the 3′ end of KRAB-ZFP genes toward their TSS, usually stopping just downstream, and no correlation between this mark and transcription levels could be established, consistent with previous observations [21]. Correspondingly, only 4 of 20 KAP1-bearing KRAB-ZFP genes were up-regulated more than 2-fold upon *Kap1* depletion in ESCs, strongly arguing against auto-regulatory loops.

In conclusion, the present work demonstrates that the bulk of KRAB-ZFP genes is expressed during early embryogenesis, paving the way to studies aimed at identifying the genomic target of specific members of this family and at delineating their functions in early development.

## Methods

### Cell Culture

Mouse ESCs and other cell types were cultured following standard conditions. For cell culture and differentiation protocols, see Supplementary Materials S1.

### Generation of the Mouse and Human KRAB-ZFP Gene Lists and Conservation Analysis

The identifiers of the “Krüppel associated box” domain of the Pfam (PF01352, http://pfam.sanger.ac.uk), InterPro (IPR001909, www.ebi.ac.uk/interpro), Prosite (PS50805, http://www.expasy.org/prosite) and SMART (SM00349, http://smart.embl-heidelberg.de) protein databases were used as filters for the Martview tool (http://www.biomart.org/biomart/martview) to identify in the NCBIM37 mouse database and in the GRCh37 human database Ensembl genes encoding for KRAB-containing proteins. The four lists of unique Ensembl gene IDs obtained with the different “Krüppel associated box” identifiers were then manually merged to form a unique list. A comparison of zinc finger sequences (fitting the pattern C-X(2–4)-C-X(12)-H-X(3–5)-H) between human and mouse KRAB-ZFPs was made using Clustal Omega version 1.1.0 using default settings; a cut-off value of 70% homology was used to build the list of homologs.

### NanoString nCounter Probe-set Library Generation

The NanoString nCounter (http://www.nanostring.com) probe-set library was generated following the instructions provided by the manufacturer [51], [52]. For each gene ID contained in our KRAB-ZFP list it was requested to design one probe-pair targeting all the known transcript variants. Genes for whom it was not possible to design specific probes that would have not hybridized also on other genes in the list had to be excluded from the analysis. The list also included 21 housekeeping genes (*Actb, Aldoa, Bak1, Cox6a1, Dkk3, Gsk3b, Hist1h1c, Hprt1, Hus1, Igf2r, Mal2, Mecp2, Rpl32, Smarcd1, Sumo1, Tbp, Tnc, Tpm4, Tubb2c, Ubl3, Ubqln2*) and 34 control genes, for which we expected specific expression patterns in the different cell types included in the analysis (*Brachyury, Cbx1, Cbx3, Cbx5, Cd3e, Cd5, cMyc, Dmd, Eomes, Esrrb, EU599041, Fbxo28, Fgf5, Gata4, Klf2, Klf4, Klf5, Lefty1, Ms4a1, Nanog, Nodal, Nr5a1, Nr5a2, Pou5f1, Ptprc, Rela, Rex1, Socs7, Sox2, Stat3, Stella, Tbx3, Tcf3, TRIM28*).

### NanoString nCounter Expression and Statistical Analysis

Total RNA was isolated from 1–10×10^6^ cells using the RNeasy Plus Mini Kit (Qiagen) following manufacturer instructions and quantified with NanoDrop. NanoString nCounter expression analysis were performed using 250 ng of total RNA per assay; each cell line was analyzed with three independent replicate assays, with the exception of the MEF cell lines for which only two replicates were performed. Following manufacturer instructions, hybridization reactions between RNA and the probe-set library were performed overnight at 65°C, before processing (loading onto cartridges and washout of excess probes) and reading by the NanoString nCounter platform [51], [52]. Raw counts for each probe-pair were first background corrected by subtracting the geometric mean of the counts obtained from negative control probes (designed to target A. thaliana genes). Negative values were corrected to 0.1. Background-corrected counts obtained from the 21 housekeeping in the 53 assays genes were then used to perform a geNorm analysis with the provided Excel macro [70] that identified *Hus1, Sumo1, Tbp and Tub2c* as the most stable ones (with geNorm values <0.5). The corresponding normalization factors for each assay were then used to normalize all the other background-corrected values. Normalized counts for each triplicate (or duplicate) were then averaged to calculate nCounter expression counts corresponding to each gene in the different cell types. Averaged normalized counts corresponding to each gene were divided by the geometric mean of the gene in all the cell types and the Log2 of this value was used for the generation of expression heat-maps and clustering analysis was performed by using the GENE-E application and the Euclidean distance between different genes (http://www.broadinstitute.org/cancer/software/GENE-E). After averaged normalized counts were divided by the geometric mean of corresponding gene in all the cell types, they were also employed to calculate the correlation coefficient of global KRAB-ZFP expression values between different samples, represented as heat-maps using the Gene-e application and hierarchical clustering analysis performed by calculating the Euclidean distance between samples. Normalized counts before average calculation were also used to perform a non-parametric statistical analysis for the identification of genes that were significantly differentially expressed (p<0.01) between the 4 subgroups: ESCs and EGCs grown in 2i+LIF conditions; ESCs and EGCs grown in BMP4/FCS+LIF conditions; EpiSCs and EBs; XEN cells, TSCs, NPCs and MEFs.

### Small-scale RNA Analysis

In order to perform real-time quantitative PCR (RT-qPCR) analysis, total RNA was extracted as described above. After quantification, 1 µg of purified RNA was reverse-transcribed to cDNA using the SuperScript II reverse transcriptase (Invitrogen) with random hexamers as primers. 1∶10–1∶100 dilutions of cDNA were used for quantification on a 7900HT Fast Real-Time PCR machine (Applied Biosystem) using SybrGreen Master Mix (Roche). Primers were designed either with the Primer Express software (Applied Biosystem) or with the resource GETPrime (http://updepla1srv1.epfl.ch/getprime [71]). For a complete list of primers, see Supplementary Materials S1. Primer specificity was confirmed by dissociation curve analysis. Normalization of qPCR data was performed on values obtained with the Actb and Tubb2c genes, unless otherwise specified. Calculation of relative quantities was performed with the ΔΔCT method, where ΔCT was calculated as the difference between CTs of specific genes and CTs of normalizer genes, and ΔΔCT was calculated as the difference between ΔCT in any sample and ΔCT in a reference sample. Relative quantities were expressed as 2-ΔΔCT, with reference samples set as 1.

### ChIP-seq Data Analysis

ChIP-seq raw data generated in Rex1-GFPd2 ESCs cultured in FCS+LIF conditions were downloaded from GSM590111 (H3K4me3), GSM590115 (H3K27me3), GSM850406 (H3K9me3) [55], GSM1032182 and GSM1032183 (KAP1) [50]. Reads were mapped to the mouse genome (mm9) using bowtie short read aligner [72] allowing up to two or three mismatches and a maximum of five repeats. Histone modification enriched regions were defined using the ChIP-Seq analysis tools web server (http://ccg.vital-it.ch/chipseq/). KAP1 ChIP-Seq peaks were defined using MACS [73] and normalised to the Total Input. Heatmaps were generated using R (http://www.r-project.org/) and Bioconductor open source packages (http://www.bioconductor.org/) and represent all annotated KRAB-ZFPs genes containing or not histone modifications and KAP1 and at their TSS (−3.5 kb +0.5 kb). To evaluate the distance between KRAB-ZFP genes and ERVs, KRAB-ZFP genes were separated in two distinct groups: those having H3K9me3 histone modification (+/−3.5 kb) and those without (192 and 143 genes respectively). Coordinates of all ERVs were downloaded from the UCSC Genome Browser (http://genome.ucsc.edu/) ERVs shorter than 500 bp were excluded from the analysis. The distance to the nearest ERV was calculated and the overall results were plotted. Statistical significance was calculated using a Wilcoxon test, with adjusted p-value <0.01.

## Supporting Information

Figure S1 **Aminoacidic alignment of the KRAB domains contained in the Ssx group of KRAB-O proteins encoded by the mouse genome.** A) HMM logo of the KRA domain adapted from the Pfam database website [8]. Red stars highlight the position of the aspartate and valine residues (D5 V6) that need to be conserved to allow the interaction between the KRAB domain and KAP1. B) Alignment generated with Clustal Omega between the KRAB domain consensus obtained from the Pfam database and the KRAB domains of the nine mouse Ssx KRAB-O proteins encoded by the genes found on cluster 50 on chromosome X. Yellow boxes highlight the position of the D5 V6 residues in the consensus sequence. Red box highlights the D5 V6 residues of the Ssx KRAB-O proteins, and absence of conservation of the V6 residue.
(TIF)

Figure S2 **KRAB-ZFP gene expression analysis correlation between NanoString nCounter platform and RNA-seq.** Expression values (NanoString counts or RPKM counts) were obtained for each KRAB-ZFP gene that was detectable above background by NanoString nCounter (counts >0.1) and RNA-seq data (RPKM counts >0) (Rowe *et al.* submitted) in ESCs grown in FCS+LIF and in MEFs. Log2 ratios between ESCs and MEFs were calculated for each gene and plotted. Correlation analysis was performed using the Prism 5 software and trend line and R^2^ values were obtained.
(TIF)

Figure S3 **Deposition of histone modifications and KAP1 enrichment over promoter regions of mouse KRAB-ZFP genes.** Heat map representing the presence (red boxes) or absence (yellow boxes) of enriched regions for the H3K4me3, H3K27me3, H3K9me3 histone modifications and for KAP1 on promoter regions of KRAB-ZFP genes. Enriched regions for H3K4me3, H3K27me3, H3K9me3 and KAP1 were identified as previously described using publicly available ChIP-seq datasets generated in mouse Rex1-GFPd2 ESCs (H3K4me3, H3K27me3, H3K9me3, [9]) and in KAP1 WT and KO ESCs (KAP1, Rowe *et al.,* submitted). Promoter regions were defined as the genomic regions 3.5 kb upstream and 500 bp downstream of KRAB-ZFP gene TSSs. Each line corresponds to a KRAB-ZFP gene, identified with its Ensembl Gene ID. Numbers following the underscore (_) represent the genomic cluster to which each gene belongs. If 0, the KRAB-ZFP gene is present in the genome as singleton.
(TIF)

Figure S4 **Binding sites of TFs of the core pluripotency network in the vicinities of pluripotency-specific KRAB-ZFP genes.** Binding sites for TFs of the core pluripotency network (CMYC, E2F1, ESRRB, KLF4, NANOG, NMYC, OCT4, SMAD1, SOX2, STAT3, ZFX) were identified using publicly available ChIP-seq data in ESCs [10]. UCSC Genome Browser representation of the genomic cluster containing Zfp459 (A) and Zfp819 (B) (both highlighted in green), and binding sites of the TFs of the core pluripotency network (black bars), showing an enrichment for binding sites in the vicinities of Zfp459 (bound by E2F1, ESRRB, KLF4, NANOG, SMAD1, SOX2, STAT3) and Zfp819 (bound by E2F1, ESRRB, KLF4, NANOG, SOX2) genomic regions, compared with the neighboring genes.
(TIF)

Table S1 **List of mouse genes encoding for KRAB-containing proteins.** The table was generated starting from unique Ensembl Gene IDs of protein coding genes predicted to encode proteins containing at least one KRAB domain. For each gene, the longest protein-coding Ensembl Transcript ID was indicated, together with the corresponding Ensembl Protein ID and aminoacidic sequence. Furthermore, it was specified the number of C2H2 ZF domains contained in each protein (0 for KRAB-O proteins) and the genomic cluster (with ascending numbers following chromosome numbers) to which the gene belongs (0 if the gene is found as singleton in the genome).
(XLSX)

Table S2 **Comparison between mouse KRAB-ZFP genes identified in this study and in previous studies** [**11**] **.** Side by side comparison between lists of mouse KRAB-ZFP genes identified in this work and in previous ones. Highlighted in red, the newly identified genes in this work; highlighted in blue, the genes previously identified that were not scored with our approach.
(XLSX)

Table S3 **List of human genes encoding for KRAB-containing proteins.** The table was generated with the same approach and criteria for Table S1. It does not contain the annotation of human genomic clusters.
(XLSX)

Table S4 **Comparison between human KRAB-ZFP genes identified in this study and in previous studies** [**12**]–[**14**] **.** Side by side comparison between lists of human KRAB-ZFP genes identified in this work and in previous ones. Highlighted in red, the newly identified genes in this work; highlighted in blue, the genes previously identified that were not scored with our approach.
(XLSX)

Table S5 **Conservation between mouse and human KRAB-ZFPs.** The table contains pairs of conserved mouse and human KRAB-ZFPs and the percentage of homology between their C2H2 ZF domains calculated using the Clustal Omega tool.
(XLSX)

Table S6 **NanoString nCounter probe-set.** The table contains Ensembl Gene IDs of mouse KRAB-ZFP genes and the corresponding NanoString nCounter probe sequences designed following the manufacturer instructions. It also contains the Gene symbols and the corresponding NanoString nCounter probe sequences of housekeeping genes included in the analysis for normalization purpose and prospectively control genes.
(XLSX)

Table S7 **NanoString nCounter expression analysis dataset.** The table contains background-corrected, normalized and averaged NanoString nCounter reads (see Methods section for details on data analysis) corresponding to all the KRAB-ZFP genes (identified with their Ensembl Gene IDs), housekeeping genes and prospectively control genes (identified with their Gene symbol) included in the probe-set, obtained from the cell types included in the analysis (see Fig. 2a). For KRAB-ZFP genes it also includes the gene coordinates and the genomic cluster to which they belong (see Table S1) to highlight the variable expression levels and behavior between different cell types in function of the chromosome positions.
(XLSX)

Table S8 **Comparison between RNA-seq and NanoString nCounter KRAB-ZFP gene expression analysis.** The table contains RPKM RNA-seq counts and background-corrected normalized NanoString nCounter counts corresponding to KRAB-ZFP genes that were detectable both in ESCs (FCS+LIF) and MEFs. These values were used to calculate the Log2 ratios between ESCs and MEFs, then used to perform the correlation analysis between RNA-seq and NanoString nCounter platform.
(XLSX)

Table S9 **Comparison between RNA-seq and NanoString nCounter KRAB-ZFP gene expression analysis.** Table indicating KRAB-ZFP genes with enriched regions for H3K4me3, H3K27me3, H3K9me3 and KAP1 determined by ChIP-seq within the regions encompassing the gene body, 3.5 kb upstream of the TSS and 3.5 kb downstream of the gene end. Coordinates indicate chromosome number and the position of the enriched regions.
(XLSX)

Supplementary Materials S1 (ZIP)
