# Supplementary figures and images for: Global and Stage Specific Patterns of Krüppel-Associated-Box Zinc Finger Protein Gene Expression in Murine Early Embryonic Cells
Source: PLoS One. 2013 Feb 22;8(2):e56721. doi: 10.1371/journal.pone.0056721 (PMC3579818; doi:10.1371/journal.pone.0056721)

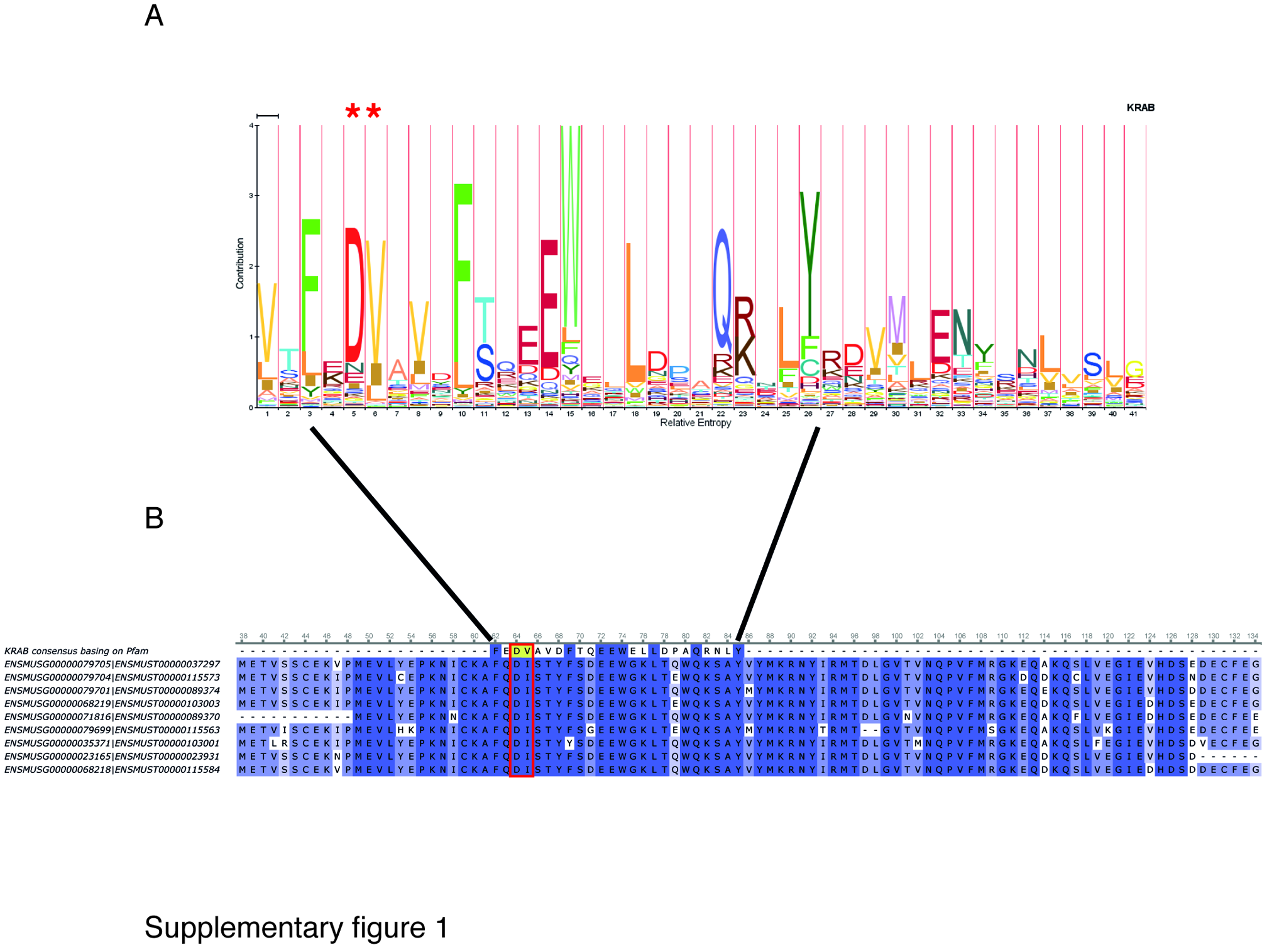

Supplement: Figure S1 — Aminoacidic alignment of the KRAB domains contained in the Ssx group of KRAB-O proteins encoded by the mouse genome. A) HMM logo of the KRA domain adapted from the Pfam database website [8]. Red stars highlight the position of the aspartate and valine residues (D5 V6) that need to be conserved to allow the interaction between the KRAB domain and KAP1. B) Alignment generated with Clustal Omega between the KRAB domain consensus obtained from the Pfam database and the KRAB domains of the nine mouse Ssx KRAB-O proteins encoded by the genes found on cluster 50 on chromosome X. Yellow boxes highlight the position of the D5 V6 residues in the consensus sequence. Red box highlights the D5 V6 residues of the Ssx KRAB-O proteins, and absence of conservation of the V6 residue. (TIF) [file pone.0056721.s001.tif]

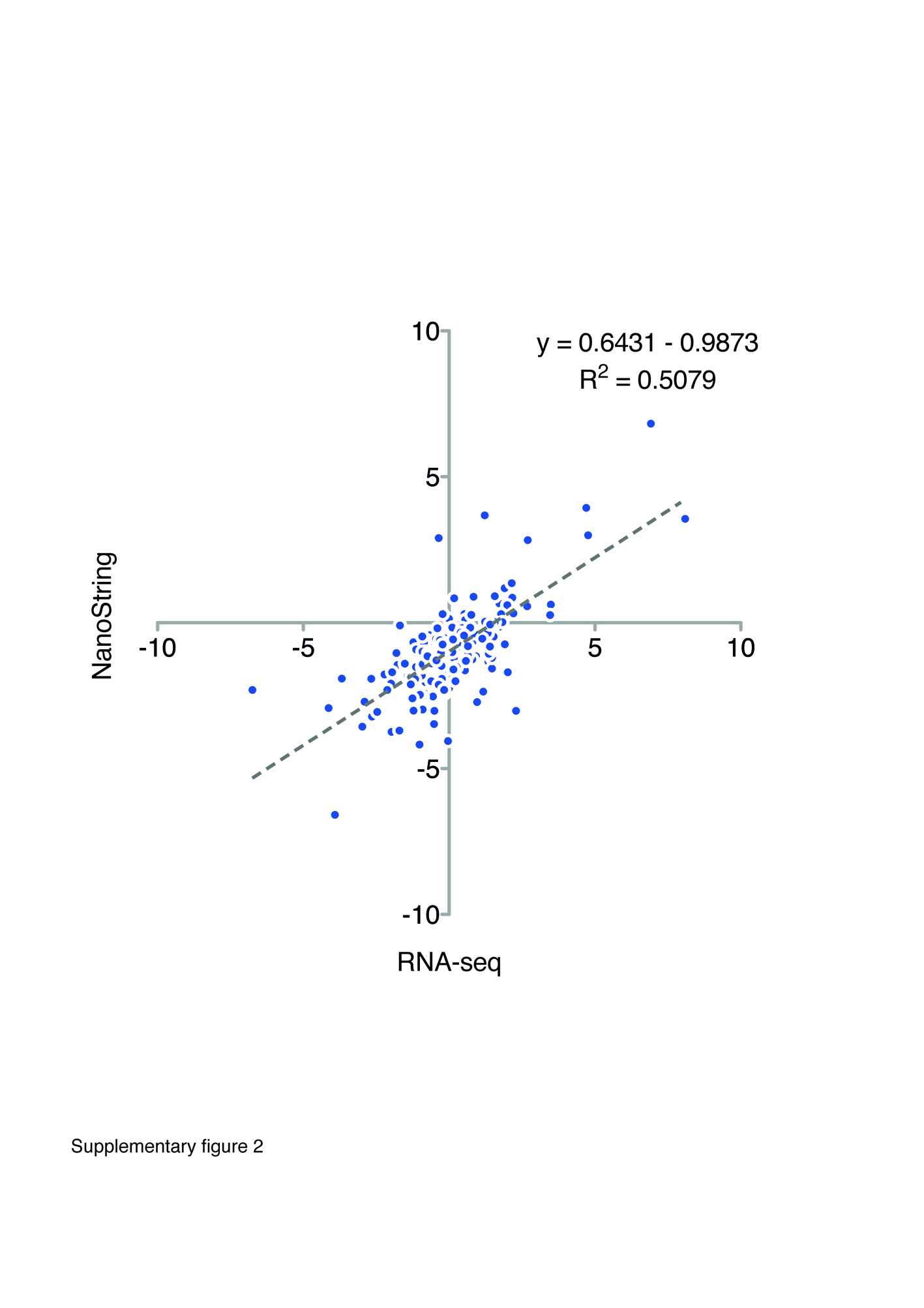

Supplement: Figure S2 — KRAB-ZFP gene expression analysis correlation between NanoString nCounter platform and RNA-seq. Expression values (NanoString counts or RPKM counts) were obtained for each KRAB-ZFP gene that was detectable above background by NanoString nCounter (counts >0.1) and RNA-seq data (RPKM counts >0) (Rowe et al. submitted) in ESCs grown in FCS+LIF and in MEFs. Log2 ratios between ESCs and MEFs were calculated for each gene and plotted. Correlation analysis was performed using the Prism 5 software and trend line and R2 values were obtained. (TIF) [file pone.0056721.s002.tif]

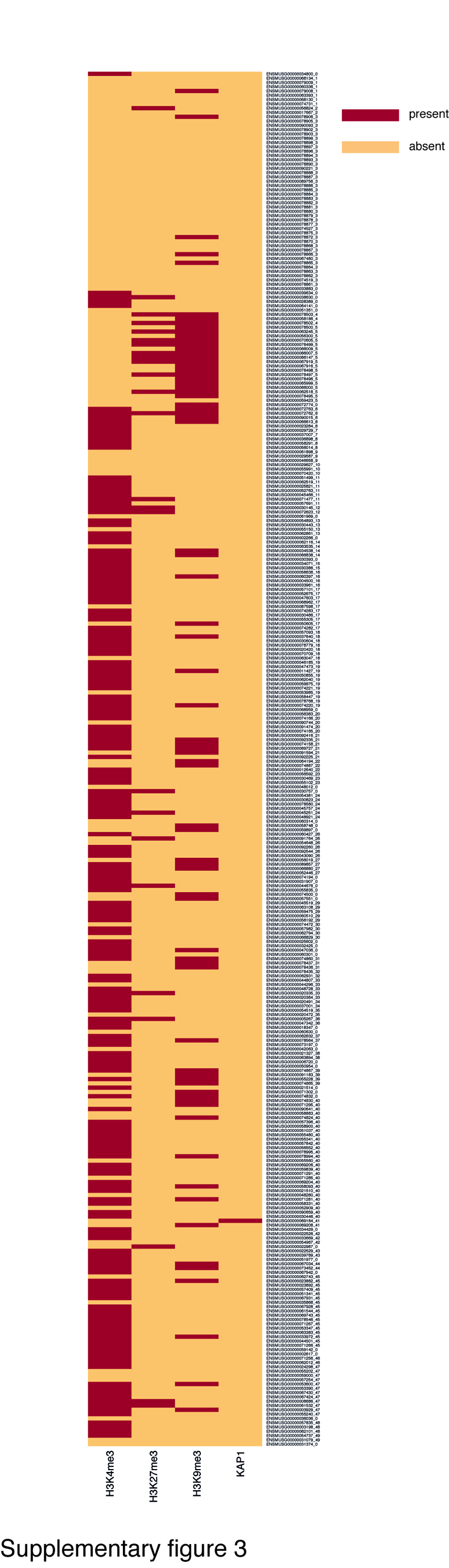

Supplement: Figure S3 — Deposition of histone modifications and KAP1 enrichment over promoter regions of mouse KRAB-ZFP genes. Heat map representing the presence (red boxes) or absence (yellow boxes) of enriched regions for the H3K4me3, H3K27me3, H3K9me3 histone modifications and for KAP1 on promoter regions of KRAB-ZFP genes. Enriched regions for H3K4me3, H3K27me3, H3K9me3 and KAP1 were identified as previously described using publicly available ChIP-seq datasets generated in mouse Rex1-GFPd2 ESCs (H3K4me3, H3K27me3, H3K9me3, [9]) and in KAP1 WT and KO ESCs (KAP1, Rowe et al., submitted). Promoter regions were defined as the genomic regions 3.5 kb upstream and 500 bp downstream of KRAB-ZFP gene TSSs. Each line corresponds to a KRAB-ZFP gene, identified with its Ensembl Gene ID. Numbers following the underscore (_) represent the genomic cluster to which each gene belongs. If 0, the KRAB-ZFP gene is present in the genome as singleton. (TIF) [file pone.0056721.s003.tif]

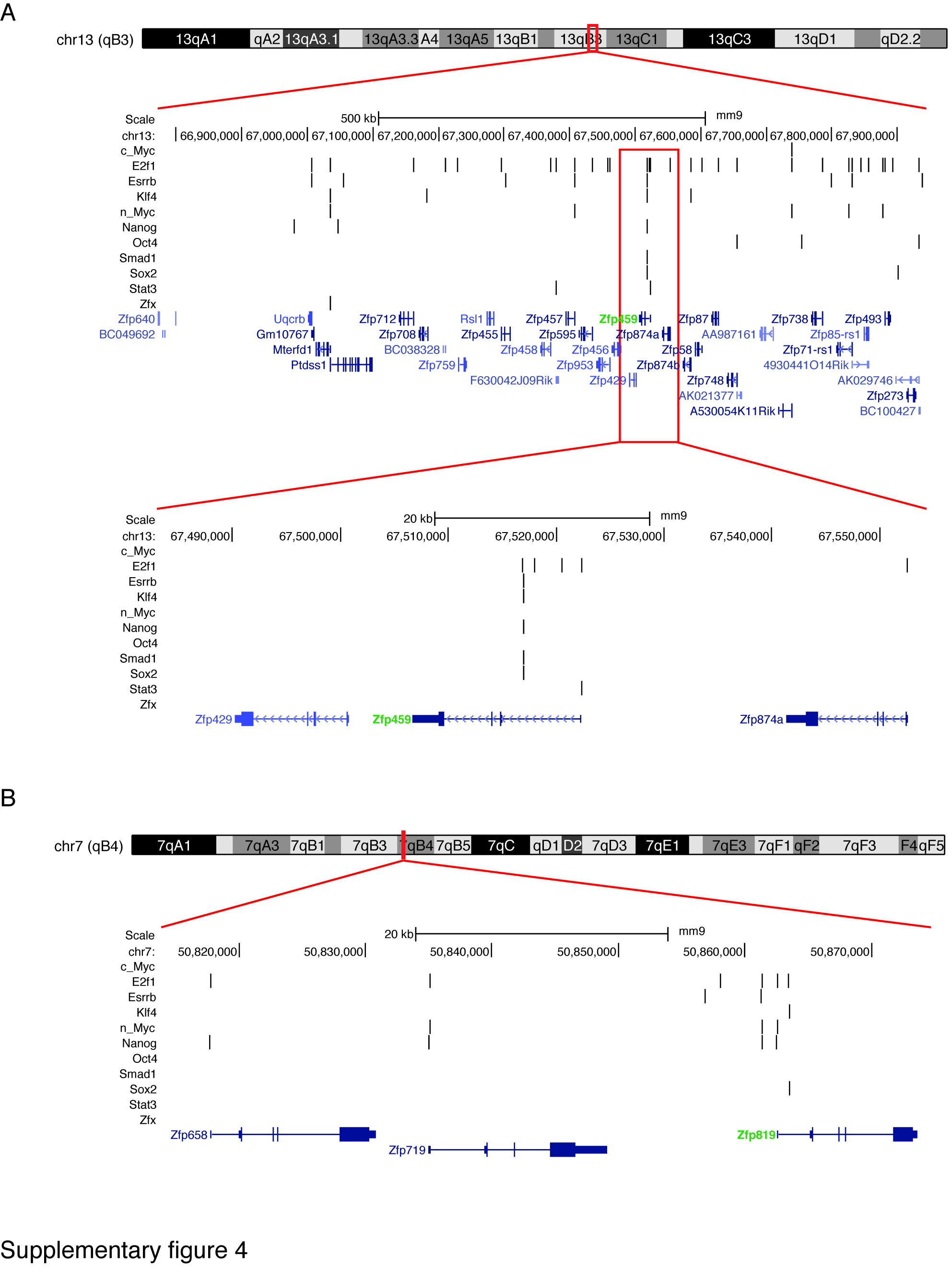

Supplement: Figure S4 — Binding sites of TFs of the core pluripotency network in the vicinities of pluripotency-specific KRAB-ZFP genes. Binding sites for TFs of the core pluripotency network (CMYC, E2F1, ESRRB, KLF4, NANOG, NMYC, OCT4, SMAD1, SOX2, STAT3, ZFX) were identified using publicly available ChIP-seq data in ESCs [10]. UCSC Genome Browser representation of the genomic cluster containing Zfp459 (A) and Zfp819 (B) (both highlighted in green), and binding sites of the TFs of the core pluripotency network (black bars), showing an enrichment for binding sites in the vicinities of Zfp459 (bound by E2F1, ESRRB, KLF4, NANOG, SMAD1, SOX2, STAT3) and Zfp819 (bound by E2F1, ESRRB, KLF4, NANOG, SOX2) genomic regions, compared with the neighboring genes. (TIF) [file pone.0056721.s004.tif]
