# Supplementary material for: Global and Stage Specific Patterns of Krüppel-Associated-Box Zinc Finger Protein Gene Expression in Murine Early Embryonic Cells
Source: PLoS One. 2013 Feb 22;8(2):e56721. doi: 10.1371/journal.pone.0056721 (PMC3579818; doi:10.1371/journal.pone.0056721)
Supplement: Supplementary Materials S1 — (ZIP) [file pone.0056721.s014.zip]

**SUPPLEMENTARY MATERIALS**

### Embryonic stem cells and embryonic germ cells

Rex1-GFPd2 ESCs [[1](#_ENREF_1)] and SLD21/22 EGCs [[2](#_ENREF_2)], a kind gift of Dr. Austin G. Smith and collaborators (Univeristy of Cambridge, UK), were cultured for less than 40 passages on 0.1% gelatin-coated culture plates. In FCS+LIF conditions, they were grown in Glasgow Modified Eagle’s Medium (GMEM, Sigma-Aldrich) complemented with 10% ES-grade FCS (PAA), Pen/Strep/l-Gln 1x (PAA), sodium pyruvate 1 mM (Sigma-Aldrich), β-ME 1mM, MEM-non-essential aminoacids 1x (Gibco) and recombinant mLIF ESGRO 1000 units/ml (Millipore). For BMP4+LIF conditions, cells were grown in N2B27 medium (Stem Cell Sciences) complemented with Pen/Strep/l-Gln 1x (PAA), recombinant mLIF ESGRO 1000 units/ml (Millipore) and recombinant hBMP4 10 ng/ml (R&D). For 2i+LIF conditions cells were grown in N2B27 medium (Stem Cell Sciences) complemented with Pen/Strep/l-Gln 1x (PAA), recombinant mLIF ESGRO 1000 units/ml (Millipore), PD032591 1 nM (Stemgent) and CHIR99021 3.3 nM (Stemgent). Cells were detached using Accutase (Gibco) at room temperature every 2-3 days and re-plated at a 1:5-1:10 dilution. Sorting of GFP^high^ and GFP^low^ Rex1-GFPd2 ESCs grown in FCS+LIF was performed using a FACSAria II cell sorter (Beckton-Dickinson).

### Epiblast stem cells

Oct4-GFP EpiSCs, a kind gift of Dr. Austin G. Smith and collaborators (Univeristy of Cambridge, UK), or ESCs differentiated into EpiSCs [[3](#_ENREF_3)] were cultured for more than 10 and less than 25 passages on plates coated with fibronectin 10 ng/ml in N2B27 medium complemented with Pen/Strep/l-Gln 1x (PAA), recombinant mActivin 20 ng/ml (R&D) and human bFGF (or FGF2) 12 ng/ml (Invitrogen).

Cells were detached using Accutase at 37°C every 2-3 days and re-plated at a 1:5-1:10 dilution.

### Mouse embryonic fibroblasts

Primary MEFs were obtained from E12.5-13.5 embryos as described in [[4](#_ENREF_4)]. Briefly, after removal of head and guts, embryos were trypsinized at 37°C for 10 minutes and plated in DMEM containing 10% FCS serum (Hyclone) and Pen/Strep/l-Gln 1x (PAA). The cells that could grow in these conditions were named MEFs and were cultured for less than 5 passages. Cells were detached using trypsin 0.25% (Gibco) at 37°C every 2-3 days and re-plated at a 1:5-1:10 dilution.

### Trophoblast stem cells and extra-embryonic endoderm stem cells

TSCs and XEN cells were a kind gift of Dr. Daniel Constam (EPFL, Switzerland) and Dr. Janet Rossant (The Hospital for Sick Children, Canada). TSCs [[5](#_ENREF_5)] were cultured in RPMI medium (Gibco) complemented with 20% FCS (Hyclone), Pen/Strep/l-Gln 1x (PAA), sodium pyruvate 1 mM (Sigma-Aldrich), β-ME 1mM, heparin 1 μg/ml (Sigma) and recombinant hFGF4 25 ng/ml (Peprotech). Cells were detached using trypsin 0.25% (Gibco) at 37°C every 2-3 days and re-plated at a 1:5-1:10 dilution.

XEN cells [[6](#_ENREF_6)] were cultured in RPMI medium (Gibco) complemented with 20% FCS (Hyclone), Pen/Strep/l-Gln 1x (PAA), sodium pyruvate 1 mM (Sigma-Aldrich), β-ME 1mM. Cells were detached using trypsin 0.25% (Gibco) at 37°C every 2-3 days and re-plated at a 1:5-1:10 dilution.

### Differentiation of ESCs

For differentiation of Rex1-GFPd2 ESCs into EpiSCs, cells were cultured in EpiSCs conditions (see above) for 18 passages. For differentiation of Rex1-GFPd2 ESCs in the absence of LIF (-LIF), cells were cultured at low density for three days on 0.1% gelatin-coated culture plates in Glasgow Modified Eagle’s Medium (GMEM, Sigma-Aldrich) complemented with 10% ES-grade FCS (PAA), Pen/Strep/l-Gln 1x (PAA), sodium pyruvate 1 mM (Sigma-Aldrich), β-ME 1mM and MEM-non-essential aminoacids 1x (Gibco). For differentiation of E14 ESCs into neural progenitor cells, a kind gift of Dr. Johan Jakobsson (University of Lund, Sweden), cells were cultured as described in [[7](#_ENREF_7)].

For generation of embryoid bodies, Rex1-GFPd2 ESCs were cultured at a density of 1-5 × 10^4^ cells/ml, for 3, 6 and 9 days on non-treated 35 mm dishes (StemCell Technologies) in Iscoves Modified Dulbecco’s Medium (Gibco) complemented with 10% FCS serum (Hyclone), Pen/Strep/l-Gln 1x (PAA), 1-Thioglycerol 1 mM (Sigma-Aldich) and 40% of methylcellulose (R&D).

**SUPPLEMENTARY REFERENCES**

1. Wray J, Kalkan T, Gomez-Lopez S, Eckardt D, Cook A, et al. (2011) Inhibition of glycogen synthase kinase-3 alleviates Tcf3 repression of the pluripotency network and increases embryonic stem cell resistance to differentiation. Nat Cell Biol 13: 838-845.

2. Leitch HG, Blair K, Mansfield W, Ayetey H, Humphreys P, et al. (2010) Embryonic germ cells from mice and rats exhibit properties consistent with a generic pluripotent ground state. Development 137: 2279-2287.

3. Guo G, Yang J, Nichols J, Hall JS, Eyres I, et al. (2009) Klf4 reverts developmentally programmed restriction of ground state pluripotency. Development 136: 1063-1069.

4. Rowe HM, Kapopoulou A, Corsinotti A, Fasching L, Macfarlan TS, et al. (2012) TRIM28 repression of retrotransposon-based enhancers is necessary to preserve transcriptional dynamics in embryonic stem cells. Genome Res.

5. Tanaka S, Kunath T, Hadjantonakis AK, Nagy A, Rossant J (1998) Promotion of trophoblast stem cell proliferation by FGF4. Science 282: 2072-2075.

6. Kunath T, Arnaud D, Uy GD, Okamoto I, Chureau C, et al. (2005) Imprinted X-inactivation in extra-embryonic endoderm cell lines from mouse blastocysts. Development 132: 1649-1661.

7. Conti L, Pollard SM, Gorba T, Reitano E, Toselli M, et al. (2005) Niche-independent symmetrical self-renewal of a mammalian tissue stem cell. PLoS Biol 3: e283.
